# Feasibility assessment of a clean and efficient fire extinguishing system for pottery jar liquor warehouses

**DOI:** 10.1038/s41598-024-64168-4

**Published:** 2024-06-11

**Authors:** Xueming Li, Wei Wan, Youkai Zhao, Gang Bai, Xunxian Shi, Bing Chen, Yutao Zhang

**Affiliations:** 1 College of Safety Science and Engineering, Liaoning Technical University, Huludao, 125105 Liaoning China; 2 Kweichow Moutai Group, Renhuai, 564501 China; 3 China Academy of Safety Science and Technology, Beijing, 100012 China; 4 Key Laboratory of Mine Thermodynamic disasters and Control of Ministry of Education (Liaoning Technical University), Huludao, 125105 Liaoning China; 5 College of Safety Science and Engineering, Xi’an University of Science and Technology, Xi’an, 710054 Shaanxi China

**Keywords:** Clean and efficient fire extinguishing technique, Liquid carbon dioxide, Water mist, Liquid nitrogen, Liquor warehouse fire, Energy science and technology, Engineering, Natural hazards

## Abstract

Clean fire extinguishing systems applicable to the pottery jar liquor warehouse are in demand. In this study, taking 53vol% liquor as the research subject, fire models of various clean fire extinguishing systems comprising water mist, liquid carbon dioxide (LCO_2_) and liquid nitrogen (LN_2_) were established using a fire dynamic simulator to determine their fire extinguishing effect. A feasibility assessment of systems was performed under different fire source types, fire source sizes, and ventilation conditions. The fire extinguishing efficiency was analyzed in terms of the fire extinguishing time, oxygen concentration, and space temperature. The results showed that the success rate of the LCO_2_ and LN_2_ fire extinguishing systems was 100%, whereas the success rate of the water mist fire extinguishing system was 95%. In terms of reducing the oxygen concentration at the bottom of the space and the temperature in the space, the LCO_2_ system exhibited the best performance, followed by the LN_2_ system, and lastly the water mist. Under different ventilation conditions and fire source types, the LCO_2_ fire extinguishing system was least affected, whereas the effectiveness of the water mist fire extinguishing system reduced under natural ventilation conditions, and the extinguishing efficiency of the LN_2_ fire extinguishing system was affected by the fire source type. Overall, the LCO_2_ system presented more advantages in extinguishing fires in pottery jar liquor warehouses and can provide a new idea for the development and application of clean and efficient fire extinguishing systems.

## Introduction

Liquor has a low flash point and is volatile, inflammable, and explosive. As a centralized storage place, a liquor warehouse has high storage capacity, frequent operation, high fire load, and rapid spreading of fires. Therefore, the safety risk is more concentrated, and the fire risk factor is high^1^. Once a liquor warehouse catches fire, it is difficult to conduct firefighting and rescue operations, and heavy casualties and property losses can be easily inflicted. For example, in November 2021, an automatic yellow rice wine warehouse caught fire in Shaoxing, Zhejiang Province, China. The net loss caused by the fire accident was approximately 16.24 million yuan. In May 2022, a fire occurred at a liquor warehouse in Renhuai, Guizhou Province, China, leading to property losses of approximately 1 million yuan. At the time, the Chinese standard GB50694-2011^2^ was restricted by economic and technological conditions; this standard is based on moutai (a grain-based liquor) and has not been updated for many years. In recent years, the fire protection design of liquor warehouses has been slightly modified in the Chinese standard^3^. However, with the increase in the varieties and production scale of liquor in China, the construction and use of large liquor warehouses by major liquor enterprises, and the development and improvement of various fire extinguishing systems, higher requirements are placed on fire prevention measures for liquor warehouses.

The fire situation in a liquor warehouse is complicated and varying. The liquor contained in a pottery jar may ignite, and the flame may only burn at the entrance of the jar (termed the jar mouth fire); or the pottery jar may crack under high-temperature heating, and the liquor may flow onto the ground, forming a pool fire; or the liquor may first exhibit different degrees of leakage and encounter an external ignition source after the formation of pool fire in different areas. If the alcohol content is above 20%, a significant fire load will be generated^4^, and an uncontrolled fire in a liquor warehouse can cause extensive damage to enterprises and society. According to a statistical survey, China’s liquor warehouses largely use water spray as the fixed fire extinguishing system, and dry powder cart and foam cart as the mobile system. Although the existing fire extinguishing system has a good fire extinguishing effect, there are evident drawbacks in the event of a fire in pottery jar liquor warehouses: On the one hand, a water spray can easily cause the high-temperature pottery jar to break under thermal stress and mechanical impact on encountering cold water, forming a flowing fire and aggravating the difficulty of extinguishing the fire. On the other hand, chemical fire extinguishing systems, such as those based on foam or dry powder, though have a better fire extinguishing effect, will pollute the liquor and increase secondary losses after fire extinguishment. Hence, it is necessary to develop new clean and efficient fire extinguishing technologies for the safe storage of liquor in pottery jars.

Since 2010, China has no longer produced or utilized Halon as a fire extinguishing agent, as it pollutes the environment^5,6^. In the past 20 years, research and development into efficient and environmentally friendly fire extinguishing technologies have become a major focus in the field of cleaner production worldwide. The water mist fire suppression technology has emerged as a strong contender. Extensive studies have been conducted on the mechanism of fire extinguishment and on the evaluation of the fire extinguishing effect of water mist^7,8^. Liang et al^9^. elucidated two mechanisms whereby ethanol fire is extinguished by water mist: flame cooling mechanism and continuous suppression until extinguishment via cooling of the fuel surface. The flash point of ethanol is low, and it is difficult to extinguish the fire source via the second mechanism. Hence, it is suggested that the water mist system can extinguish ethanol fires directly through the first mechanism as much as possible. The extinguishing mechanism of the water mist extinguishing system depends on the type of fire^10^. Its fire extinguishing effect is subject to the characteristics of the water mist^11,12^, nozzle^13,14^, ventilation condition^15,16^, and other factors. Currently, the research object in experiments on the water mist fire extinguishing effect is mostly ethanol pool fire, and few scholars have studied the effectiveness of the water mist fire extinguishing system in the case of fires in liquor warehouses. Although the concentration of most ethanol combustibles considered in previous experiments was greater than that in general liquor, based on Chen’s^17^ research on the differences in the heat release rate (HRR) and flash point between ethanol–water mixture and Chinese liquor, the overall difference between the ethanol–water mixture and liquor is not significant, and the experimental results pertaining to the ethanol–water mixture can be used to predict the combustion characteristics of Chinese liquor. The feasibility of the water mist system in extinguishing fires in pottery liquor warehouses can be preliminarily assessed from related research on the application of this system in extinguishing ethanol pool fires. However, its extinguishing mechanism and extinguishing efficiency are restricted by many factors. Zhou et al.^18^ conducted extinguishing experiments on ethanol pool fires under the coupling effect of the water mist system and mechanical exhaust at different exhaust rates. The experimental results showed that an ethanol pool fire cannot be suppressed thoroughly by water mist. Luo et al.^19^ applied water mist to an ethanol fire for 150 s and found that the fire could not be extinguished. However, after the addition of inert gases to extinguish the fire, the extinguishing effect was remarkable, and the extinguishing times for CO_2_ and N_2_ were 70 s and 93 s, respectively. The main reason is that an inert gas plays a key role in reducing the volume fraction of the oxygen. An inert gas (CO_2_ and N_2_), which is a green, clean fire extinguishing agent with asphyxiation as the main extinguishing mechanism, has the advantages of high extinguishing efficiency and speed^20^. CO_2_ and N_2_ can extinguish liquid and other types of fires, making them suitable for factories and warehouses containing more combustible materials, and will not cause damage to the liquor.

Considering the compressibility limits of gaseous CO_2_ and N_2_, liquid carbon dioxide (LCO_2_) and liquid nitrogen (LN_2_) can be used as alternatives to store a larger amount of fire extinguishing media, which is conducive to the rapid and large-volume injection into the liquor warehouse. In addition, the nozzle releases CO_2_ (N_2_) in the gas–liquid mixed state, and the low temperature inert gas exchanges heat with the air in the space, absorbing a lot of heat and effectively reducing the temperature of the fire site^21,22^. Song et al.^23^ proposed a treatment method for thermal disaster problems using LCO_2_ as the cooling source. Ground tests were conducted through a self-designed LCO_2_ phase-change refrigeration and cooling device. The experimental results showed that the utilization rate of the LCO_2_ cooling volume was 77.36%, and an effective cooling could be achieved. Liang et al.^24^ conducted a cooling and fire prevention experiment with LN_2_ injection to treat high-temperature burning coal in a closed chamber space. The results showed that LN_2_ injection had an evident effect on reducing the temperature of the high-temperature coal and could achieve the purpose of rapid cooling and fire prevention. However, fire extinguishing systems with LCO_2_ and LN_2_ as the inert gases are mainly used in relatively closed spaces, such as mines and cabins, where they have been demonstrated to achieve a good fire prevention effect^25–28^. There are no studies or cases on the use of these systems in liquor warehouses, and their fire extinguishing effect remains unclear. Therefore, it is of great significance to study the application of LCO_2_ and LN_2_ in clean and efficient fire extinguishing systems intended for pottery jar liquor warehouses and clarify their fire extinguishing effect.

In this study, a fire dynamic simulator (FDS) was used to perform a feasibility test on fire extinguishing systems for pottery jar liquor warehouses, so as to provide a new development direction for a green, clean, and efficient fire extinguishing technology for liquor warehouses and to improve the level of fire prevention and control.

## Model building and grid division

**Figure 1:**
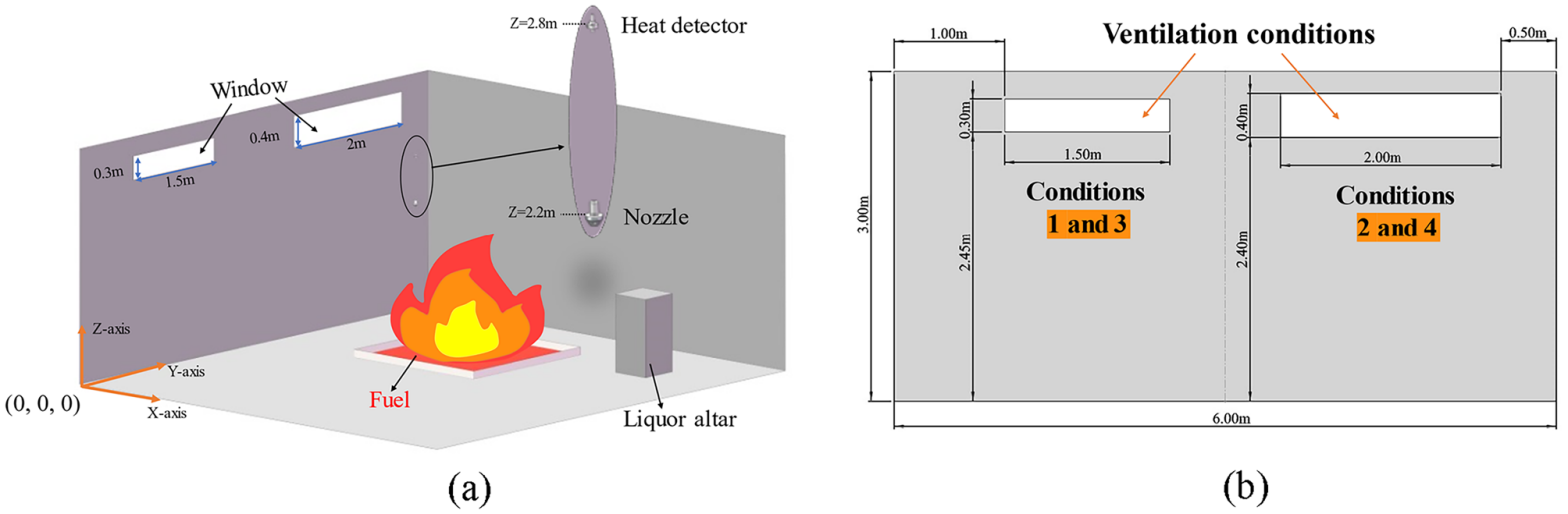
Fire extinguishing system model of liquor warehouse. (**a**) Diagram of model (hide part of walls). (**b**) Diagram of ventilation conditions.

Taking a pottery jar liquor warehouse in Renhuai area of Guizhou Province that caught fire as an example. a simplified fire model was established based on the site situation of the liquor warehouse building. The FDS was utilized to perform a computer simulation of a single fire protection zone in the liquor warehouse, as shown in Fig. 1, which shows a diagram of the physical model of the liquor warehouse. As shown in Fig. 1a, the model dimensions were 6.5 m (X direction) × 6 m (Y direction) × 3 m (Z direction). Based on a mesh sensitivity study^29^ and a comprehensive consideration of the computation time for the simulation, a grid with dimensions of 0.1 m × 0.12 m × 0.1 m was deemed reasonable and was adopted. A total of 97,500 grids were created.

The simulated ambient temperature was 20 °C, the ambient pressure was 1.013 Pa, and the mass fraction of ambient oxygen was 0.232378 kg/kg (the volume fraction is about 21%). The simulation ran for 600 s. The HRR is a key parameter to describe the fire process, and its value determines whether the simulation is consistent with the actual situation. Depending on the measurement of the physicochemical property data of high alcohols in the early stage, the specify heat of combustion of 53 vol% liquor was set to 29,636 kJ/kg, and the HRR was set to 455 kW/m^2^ ^17^. A medium-speed fire was selected in the “T-square” fire-growth model^30^. Table 1 presents the simulation conditions of the fire source. In the simulation, the starting of the fire extinguishing system was controlled by a heat detector. When the heat detector reached a temperature of 65 °C, the fire extinguishing system was activated after a delay of 20 s. The nozzle was placed at (x, y, z) = (3.5 m, 3 m, 2.2 m), and the heat detector was placed at *Z* = 2.8 m directly above the nozzle. The oxygen sensor was placed at *Z* = 0.3 m directly below the nozzle to monitor the changes in the oxygen content at the bottom of the space. To consider the effect of the pottery jar in an authentic liquor warehouse on the effectiveness of the fire extinguishing system, the pottery jar was simplified as a cubic obstacle with dimensions of 0.5 m × 0.5 m × 1 m.

**Table 1:** Simulate conditions of fire source.

Fire source	Type of fire source	Fire size (length × width)	Fire center position (x, y)	Total area (m^2^)	HRR (Kw)
1	Jar mouth fire	0.5 m × 0.5 m	(2.25 m, 4.75 m)	0.25	113.75
2	Pool fire	2 m × 1 m	(2 m, 4.75 m)	2	910
3	Pool fire	2 m × 3 m	(2.1 m, 4.5 m)	6	2730
4	Pool fire	3 m × 4 m	(2.5 m, 4.1 m)	12	5460

Three common fire extinguishing systems comprising water mist, LCO_2_, and LN_2_ were selected to find an optimal fire extinguishing system suitable for liquor warehouses. To ensure the reliability of the fire extinguishing system, the fire extinguishing efficiency under the most unfavorable conditions was simulated, that is, fuel depletion was not considered in the simulation. Because the liquor warehouse uses normally closed fireproof doors, the sealing is better, and the amount of CO_2_ leaked from small openings such as door gaps is ignored when injecting CO_2_. In order to further explore the fire extinguishing effect of the above system under different fire sources and natural ventilation conditions. Based on the actual height of the shutters on site, natural vents of different sizes were created on both sides of the model. The sizes and positions of openings in ventilation conditions 1 and 3 were shown as the left window in Fig. 1b, and the right window was shown as ventilation conditions 2 and 4. The simulated ventilation conditions are shown in Table 2.

**Table 2:** Simulate conditions of ventilation.

Ventilation	Opening size (length × height)	Total opening area (m^2^)	The state of the windows	LCO_2_ design flow rate (L/min)
1	/	0	Normally off	134.73
2	1.5 m × 0.3 m	1.8 m^2^	Normally open	147.76
3	2 m × 0.4 m	3.2 m^2^	Normally open	157.86
4	1.5 m × 0.3 m	1.8 m^2^	Open first, then close	134.78
5	2 m × 0.4 m	3.2 m^2^	Open first, then close	134.78

“/” denotes that totally-enclosed. “Open first, then close” denotes that the windows are open, close the windows when the fire extinguishing system is activated.

Design the minimum design flow and nozzle pressure for each extinguishing system in accordance with the outlined in the code of design:

1. The high-pressure water mist system has a greater impact pressure, which is likely to cause the fracture of the pottery altar and increase the fire risk, hence the low-pressure water mist fire extinguishing system^31^ is chosen as the research object, and the nozzle pressure was set to 1.2MPa. The flow rate is calculated as 78L/min according to Eq. (2). The size of the water mist that effectively extinguishes the ethanol fire ranges from 200 to 400 µm^32,33^, based on the comprehensive consideration of the size and location of the fire source, the particle size of the water mist was set to 400µm, atomization angle was set to 120°;
  1 $$N = \frac{SW}{q}$$

Here, *q* (L/min) is the flow rate of the nozzle,* S* (m^2^) is protected area of a protected object,* W* (L/min·m^2^) is the minimum design supply intensity of protected objects, is 2 L/min·m^2^; *N* is number of nozzles, was 1; Therefore, Eq. (1) was replaced by Eq. (2).

2 $$q = SW$$

(2) The nozzle pressure of LN_2_ fire extinguishing system is set to 1MPa according to the design specification of nitrogen gas fire extinguishing system^34^. The design dosage is calculated according to Eqs. (3)–(5), maximum discharge time are 60 s, the minimum design flow rate was set as 99.89L/min;
  3 $$M_{{LN_{2} }} = M_{{\text{x}}} + K \cdot \frac{V}{S}\ln \left( {\frac{100}{{100 - C}}} \right)$$
  4 $$M_{{\text{x}}} = \frac{4.041V}{{273.15 + T}}$$
  5 $$S = 0.79968 + 0.00293T$$

Here, $M_{{LN_{2} }}$ (kg) is the design dosage of N_2_; *M*_x_ (kg) is opening-compensation, it can be calculated by Eq. (4); *K* is altitude correction factor of protected area, is 1; *V* (m^3^) is net volume of protected area; *S* (m^3^/kg) is the specific volume of nitrogen, it can be calculated by Eq. (5); *T* (°C) is expected minimum temperature in the protected area, is ambient temperature,20 °C; *C* (%) is the design concentration of fire extinguishing in the protected area, is 44.85;

(3) Because the application mode of the system is total flooding extinguishing system. The nozzle pressure of the LCO_2_ fire extinguishing system was set to 1.4MPa in reference to the design specification for high-pressure CO_2_ gas fire extinguishing systems^35^. Because the design dosage of CO_2_ fire extinguishing agent is related to the size of the opening area^35^, the design flow of the LCO_2_ system was calculated by Eqs. (6)–(8).
  6 $$M_{{LCO_{2} }} = K_{{\text{b}}} (K_{1} A + K_{2} V)$$

Here, $M_{{LCO_{2} }}$ is the design dosage of CO_2_, kg; *K*_b_ is material factor, is 1.34; *K*_1_ is area factor, is 0.2kg/m^2^; *K*_2_ is volume factor, is 0.7kg/m^3^; *A* is reduced area, m^2^; *V* is net volume of protected area, m^3^.

*A* can be calculated by Eq. (7), where *A*_*v*_ is the total area of the inner side, bottom and top surfaces (including the openings therein) of the protection area, m^2^; *A*_*o*_ is the total area of the opening, m^2^. *V* can be calculated by Eq. (8), Where *V*_*v*_ is the volume of the protection area, m^3^; *V*_*g*_ is total the volume of non-combustible and difficult-combustible in the protected area, m^3^.

7 $$A = A_{{\text{v}}} + A_{{\text{o}}}$$

8 $$V = V_{{\text{v}}} - V_{{\text{g}}}$$

Under the working conditions of the model in the manuscript, the approximate calculation formula of the design flow of LCO_2_ can be simplified to Eq. (9), and the minimum design flow of the LCO_2_ system under different opening areas can be calculated according to the injection time of 1min, as shown in Table 2.

9 $$y = 134.78 + 7.211x$$

Here, y is the design flow rate of LCO_2_, L/min, x is the total area of the opening, m^2^.

**Figure 2:**
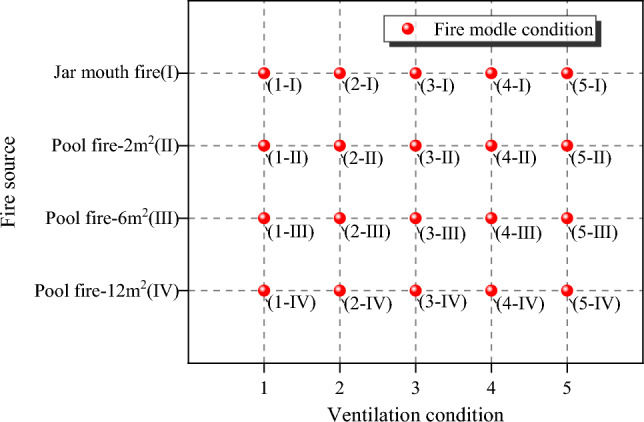
Summary of fire models under the intersection of ventilation conditions and fire source types.

The fire extinguishing effect under different fire extinguishing system conditions was tested under four different fire sources (Table 1) and five different ventilation conditions (Table 2). Under the combination of two factors, a total of 20 different fire model conditions were simulated, as shown in Fig. 2. For example, Condition 2-III refers to the fire model under ventilation condition 2 (normally open: 1.8 m^2^) and fire source III (pool fire: 6 m^2^). A total of 80 liquor warehouse fire models without any extinguishing system, water mist, and LCO_2_ and LN_2_ fire extinguishing systems were evaluated.

## Results and discussion

### Comparison between fire extinguishing systems in terms of the fire extinguishing time

**Figure 3:**
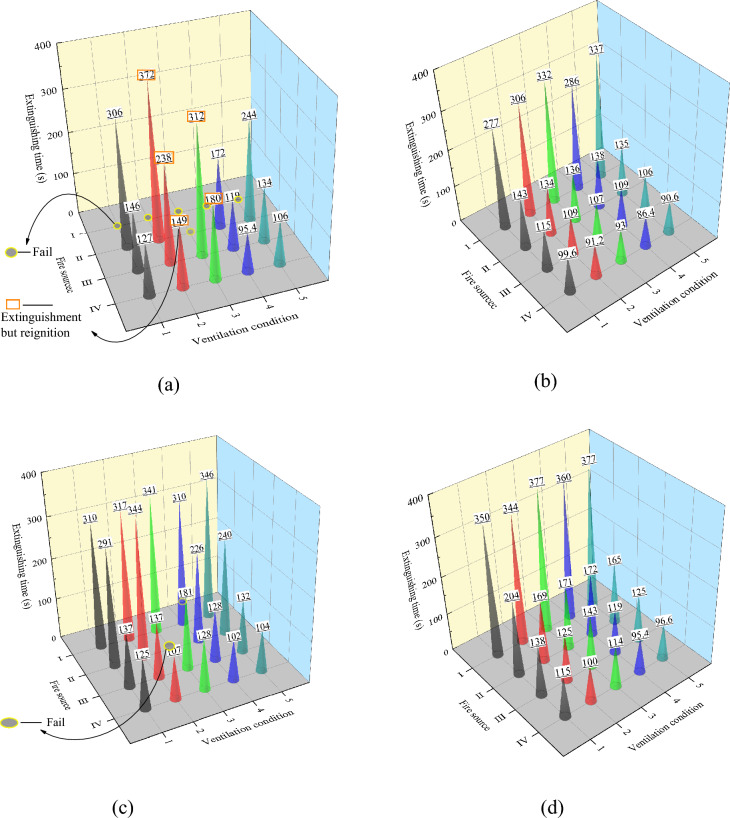
Comparison of extinguishing time under the action of various fire extinguishing systems. (**a**) No extinguishing system. (**b**) LCO_2_. (**c**) Water mist. (**d**) LN_2_.

The fire extinguishing time is an important index to measure the effectiveness of a fire extinguishing system. Figure 3 shows the time required to extinguish a simulated liquor warehouse fire under the action of various fire extinguishing systems, in which “Extinguishment but reignition” refers to the phenomenon of burning again after the HRR of the fire source briefly becomes 0 during the simulation process, in fact, this represents a failure to extinguish the fire (the data in Fig. 3 show the first extinguishment time). “Failure” means that the fire source remains in a continuous burning state during the simulation duration (600 s). Among the 20 fire models without the action of the fire extinguishing system, a total of six models failed to extinguish the fire, another five of the models could extinguish the fire but it reignited, and the remaining nine models could automatically extinguish the fire due to the insufficient oxygen in the space. These results are consistent with previous literature^36^ in that if the fire size exceeds the critical value determined by the conditions in the confined space, the fire can still be extinguished without the action of the fire extinguishing system. The application of the three fire extinguishing systems had an evident suppression effect on the fire source. In terms of the fire extinguishing result, 1 of the 20 fire extinguishing models of the water mist system had failed, and the fire extinguishing success rate was 95%. Under the action of the LCO_2_ and LN_2_ fire extinguishing systems, there was no fire extinguishing failure, and the extinguishing success rate of the fire source was the highest, which was 100%. However, the time required to extinguish the fire source by the different fire extinguishing systems was quite different under the fire source and ventilation conditions.

Different fire extinguishing types are the main reasons for the influence of fire source on the difference in the fire extinguishing time. When the fire source type is pool fire, LCO_2_ has the highest extinguishing rate, followed by LN_2_, and lastly the water mist. Under the action of the three fire extinguishing systems, the extinguishing time of the fire source advances with the increase in the intensity of the fire source. This is mainly because when the area of the fire source increases, the oxygen consumption and heat production increase, and the lack of oxygen in the space and the early start of the fire extinguishing system will prolong the time required to extinguish the fire source. The greater the area of the pool fire, the lower the difference between the fire extinguishing time results between the fire extinguishing systems. For example, under Condition 5-III (pool fire: 6 m^2^), the fire extinguishing times for LCO_2_ were 19 s and 26 s faster than those for the LN_2_ and water mist systems, respectively. Under Condition 5-IV (pool fire: 12 m^2^), the fire extinguishing times for LCO_2_ were 6 s and 13.4 s faster than those for the LN_2_ and water mist systems, respectively. The high amount of oxygen consumed by the large-area fire source will weaken the performance of fire extinguishing systems based on the asphyxiation and inerting of the fire source. Nevertheless, under the maximum fire source area of 12 m^2^, the LCO_2_ system exhibited the earliest extinguishing time.

When the fire source type is a jar mouth fire, due to its low HRR and plume mass, the temperature of the heat detector rises gradually, and the starting time of the fire extinguishing system is greater than 260 s. Thus, the overall extinguishing time of this fire source is greater than that of the pool fire. In terms of the extinguishing time for the jar mouth fire, the LCO_2_ system is still the fastest, but in contrast to the pool fire, the extinguishing rate of the water mist system is higher than that of the LN_2_ system. For example, under Condition 5-I, the extinguishing time for LCO_2_ is 337 s, and the corresponding values for the water mist and LN_2_ are 346 s and 377 s, respectively. The LCO_2_ system can extinguish the fire 9 s and 40 s faster than the water mist and LN_2_ systems, respectively. This is mainly because the inert gas can easily spread in space, and in the face of a small area of fire (jar mouth fire), the effect of cooling the fire source and isolating the oxygen near the fire source will have a delayed effect. The pertinence of its inhibiting effect on the jar mouth fire is worse than that of water mist. However, the extinguishing time under the action of the LCO_2_ extinguishing system is earlier than that of the water mist.

This is mainly because the fire area of the jar mouth fire is only 0.25 m^2^, and its low HRR leads to a relatively low fire source temperature, and the heat received by the water mist is reduced. The longitudinal distance between the nozzle and the fire source changes from 2.2 m under the pool fire to 1.2 m, and the time for which the water mist contacts the high-temperature fire source is shorter. Therefore, when the fire source type is the jar mouth fire, the particle size of 400 µm water mist is greater, which makes the droplet to receive less heat and transfer it more quickly, resulting in the evaporation of the water mist, yielding unsatisfactory results and limiting its role in displacing oxygen near the fire source. At this time, when the water mist fire extinguishing system extinguishes the jar mouth fire, it belongs to the second situation described in literature^9^, i.e., the main fire extinguishing mechanism is fuel surface cooling. Due to the horizontal offset between the mouth of the liquor pottery jar and the nozzle, fewer droplets can effectively enable the surface cooling of the fuel source, and the other water droplets that do not reach the fire source area do not absorb heat or undergo a phase change, which significantly reduces the fire extinguishing cooling efficiency of the water mist fire extinguishing system. With regard to the low-temperature CO_2_ sprayed by the LCO_2_ fire extinguishing system, the low-temperature CO_2_ will spread in space. Although the targeting of the fire extinguishing gas to the fire source is reduced in the initial start-up, the LCO_2_ fire extinguishing system is slightly faster than the water mist in terms of the final extinguishing time owing to its best cooling capacity and oxygen isolation effect among the three fire extinguishing systems.

The difference in the space tightness is the main reason for the influence of ventilation conditions on the difference in the fire extinguishing time. Moreover, the effect of space tightness on the extinguishing times of the different fire sources is quite different, and the effect of space tightness is the greatest when the fire source type is the jar mouth fire. For example, under Conditions 2-I, 3-I, and 4-I, the size of the opening before the start of the fire extinguishing system is different, and the opening is in different closed states after the start of the fire extinguishing system. Different ventilation conditions lead to differences in the space tightness. Under the three working conditions, the extinguishing times of the fire source for the LCO_2_ system were 306, 332, and 286 s, respectively. The extinguishing times of the fire source under the water mist system were 317, 341, and 310 s, respectively. The extinguishing times of the fire source under the LN_2_ system were 344, 377, and 360 s, respectively. The three fire extinguishing systems all have the longest extinguishing time under working Condition 3-I. This is because the space tightness under this condition is the worst, and a larger opening area will suck up more fresh air before the fire extinguishing system is started. Natural ventilation continuously provides fresh air during the extinguishing process. Therefore, improving the space sealing performance can further improve the inerting effect of extinguishing the fire. For example, the extinguishing time required by the water mist fire extinguishing system for the fire source in a confined space (working condition 4-I) is 17 s shorter than that in a natural ventilation environment (working condition 2-I). This is mainly because the jar mouth fire consumes less oxygen, and the better space sealing performance prevents the surrounding environment from providing fresh air, which is conducive to controlling the oxygen supply to the fire source, thus promoting the fire source to extinguish. However, in particular, the LCO_2_ fire extinguishing system considers a more open compensation flow in a naturally ventilated environment to ensure the inerting effect in the space. For example, the design flow rates were 147.76, 157.86, and 134.78 L/min under working Conditions 2-I, 3-I, and 4-I, respectively. However, the increased design flow had little influence on the extinguishing effect of the jar mouth fire, and the extinguishing time under the three working conditions showed an opposite trend to its design flow, which was positively correlated with the space tightness. This shows that the extinguishing time is more affected by the space tightness when LCO_2_ is used to extinguish the jar mouth fire.

When the fire source type is pool fire, the three fire extinguishing systems in the closed space (ventilation conditions 1, 4, and 5) have similar extinguishing rates under different fire source areas. This mainly indicates that the water mist system is more likely to extinguish large-area fire sources in a confined space, consistent with previous experimental results^37,38^. The extinguishing of the fire source is the result of a decrease in the oxygen concentration in the space caused by the consumption of oxygen by the fire source and the dilution of the oxygen by the high amount of steam generated. The inert gases produced by the vaporization of LCO_2_ and LN_2_, under the action of oxygen consumption and displacement, also meet the regulation that large-area fire sources are easier to extinguish in confined spaces. However, under natural ventilation (ventilation conditions 2 and 3), the three fire extinguishing systems have slightly different fire extinguishing performances. Both LCO_2_ and LN_2_ can effectively extinguish the fire. However, the fire extinguishing effect of the water mist was not ideal. This mainly shows that the fire extinguishing effect of the water mist decreases with the increase in the opening area. For example, when the fire source was a pool fire with an area of 6 m^2^, it could be extinguished in 137 s when the opening area was 1.8 m^2^, and the first extinguishing time was delayed to 181 s when the opening area was increased to 3.2 m^2^. When the fire source was a pool fire with an area of 2 m^2^, while the opening area was increased from 3.2 m^2^, the extinguishing time changed from 344 s to failure. This is because the actual effect influencing the oxygen consumption and displacement in the application of the water mist system depends on the HRR of the fire, confined space volume, and ventilation conditions^39^. That is, when the opening area is large, the external enrolling and infiltration of fresh air increases, the effect of water mist on displacing oxygen becomes worse, and the fire extinguishing time is delayed. At this time, compared with the pool fire with other areas, the pool fire of 2 m^2^ has the least an HRR and oxygen consumption per unit. Therefore, under a small area (2 m^2^) of pool fire, the fire extinguishing effect of the water mist fire extinguishing system is more evidently affected by the increase in the opening area.

### Variation in the oxygen concentration at the bottom of the space

**Figure 4:**
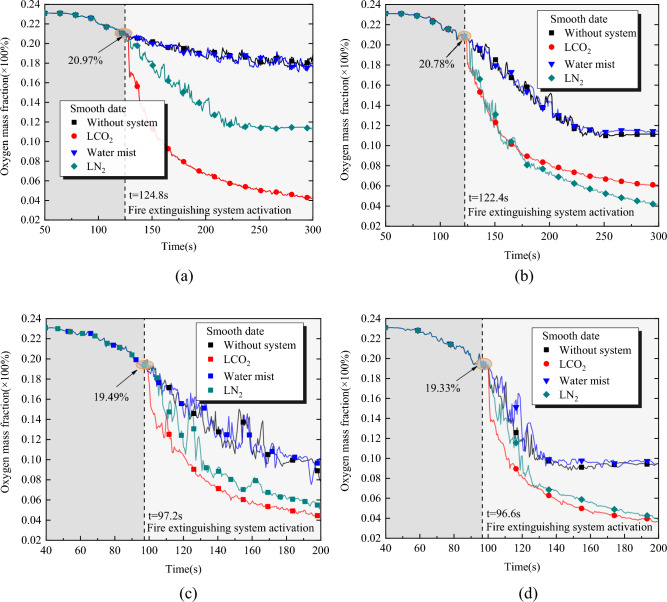
Variation of oxygen concentration at the bottom of space under different fire extinguishing systems. (**a**) Condition 3-II (Normally open3.2m^2^/pool fire-2m^2^). (**b**) Condition 4-II (Open first, then close-1.8m^2^/pool fire-2m^2^). (**c**) Condition 3-III (Normally open-3.2m^2^/pool fire-6m^2^). (**d**) Condition 5-III (Open first, then close-3.2m^2^/pool fire-6m^2^).

The oxygen content not only plays a crucial role in the alteration of combustion sources but also serves as a significant parameter for characterizing the fire suppression ability of inert gas extinguishing agents. Based on the analysis made in “Comparison between fire extinguishing systems in terms of the fire extinguishing time” section, to clearly explore the ability of each fire extinguishing system to displace oxygen near the fire source during a fire in a liquor warehouse, in this section, the changes in the oxygen concentration of small-area fires (pool fire: 2 m^2^ and pool fire: 6 m^2^) are mainly discussed. Figure 4 shows that the variation in the oxygen concentration before the fire extinguishing system is the same. Under the same fire source area, as shown in Fig. 4a,b, the larger the opening, the slightly greater the oxygen content of the fire extinguishing system when starting. Figure 4a,c show that under the same opening area, when the fire extinguishing system is started, the larger the fire source area, the lower the oxygen content. These phenomena are in accordance with the conclusions drawn from the theoretical analysis in that the external enrolling and infiltration of fresh air increase with the increase in the opening area, and the oxygen consumption increases with the increase in the fire area.

Based on the decrease in the O_2_ mass fraction of the model without the extinguishing system, it can be found that the decrease rate and amount are mainly caused by the combustion oxygen consumption and the ventilation condition. In the case of large-area fire sources, the O_2_ mass fraction decreased faster and more rapidly, and the amount of decrease is greater (the decrease seen in Fig. 4c is faster than that in 4a). Under the ventilation condition of open first and then close, the O_2_ mass fraction drops faster (Fig. 4a–d).

Under the action of the fire extinguishing system, the inerting effect of the fire extinguishing system will further affect the rate and amount of O_2_ decline. Among the three fire extinguishing systems, the water mist system exhibited the lowest performance in isolating O_2_ at the bottom of the space, and the change in the O_2_ content was slightly different from that under the action of the no-fire extinguishing system. This is mainly because when the water mist contacts the combustion material or flows through the combustion zone, it expands the vaporized volume of the water mist through heat conduction, forming a water vapor to achieve isolation of the oxygen from the fire source. However, its asphyxiation effect is mainly reflected in reducing the oxygen concentration in the combustion zone, and its inerting effect in the non-ignition region is worse than that of the inert gas extinguishing agent.

In the early stages of activation of the fire extinguishing system, the LCO_2_ system exhibited the most rapid reduction in the oxygen content, followed by the LN_2_ system. With the continuous injection of the fire extinguishing agent, the mass fraction of O_2_ also continued to decrease. Next, the spatial bottom inerting effect of LCO_2_ and LN_2_ depending on the type of pool fire was analyzed. Taking the pool fire-2m^2^ as an example, under Condition 3-II, the LN_2_ system reduced the O_2_ mass fraction to approximately 11% when *t* = 300 s. However, under Condition 4-II with better spatial tightness, the O_2_ mass fraction dropped to 4.2%. This is because the opening area under working Condition 4-II was smaller and jointly controlled closed the opening when the fire extinguishing system was started, providing a good airtight space. This significantly increased the decrease amount and rate of O_2_ at the bottom of the space and ensured the inerting effect at the bottom of the space. However, the inerting effect of the LCO_2_ and LN_2_ fire extinguishing systems showed opposite change rules under the two working conditions. Under the action of the LCO_2_ system, the O_2_ mass fraction at the bottom of the space decreased to approximately 4.2% under Condition 3-II, but only to 6% under Condition 4-II, which was worse than that under the action of the LN_2_ system. This is mainly because in a natural ventilation environment, the design flow rate of the LCO_2_ fire extinguishing system increased with the opening size to 157.86 L/min (Condition 3-II), which better ensured the inerting effect of the LCO_2_ fire extinguishing system under the natural ventilation condition. The design flow rate of LCO_2_ under the working condition 4-II was 134.78 L/min, and the decrease in the design flow rate was the main reason for the decrease in O_2_ at *t* = 300 s under Condition 4-II. In terms of the vaporization expansion ratio of LCO_2_ and LN_2_ per unit volume, the expansion volume multiple of the CO_2_ gas was lower than that of N_2_. Therefore, the performance of LCO_2_ in isolating oxygen at the bottom of the space after approximately 175 s was worse than that of LN_2_ under Condition 4-II (Fig. 4b). However, the fire extinguishing effect of the LCO_2_ fire extinguishing system was unaffected by the design flow reduction under the better sealing condition 4-II. Under Condition 4-II, the extinguishing time of the fire source under the action of LN_2_ was 172 s, which was greater than that of the LCO_2_ system (138 s). This is mainly because LCO_2_ has a higher vaporization rate than LN_2_, which makes the O_2_ concentration to drop fastest in the LCO_2_ system at the initial start-up of the fire extinguishing system, and the relationship between the specific gravity of the main gases in space is as follows: CO_2_ > O_2_ > N_2_. Among the three gases, CO_2_ is more likely to migrate to the bottom of the space and accumulate. On the contrary, N_2_ tends to move and accumulate at the middle and upper parts of the space. With the continuous injection of the fire extinguishing agent, N_2_ with an upward migration tendency causes a change in the oxygen concentration at the bottom of the space to fluctuate significantly. Consequently, the decreasing rate of the oxygen concentration at the bottom of the space reduces, the stability of the inerting effect becomes worse, and the fire extinguishing time is prolonged.

A liquid inert gas forms a large amount of inert gas through phase transition vaporization. Thus, there is a rapid injection of a large amount of inert gas into the fire area, so that the oxygen concentration in the space can be rapidly reduced, playing a role in inhibiting the fire. The space bottom inerting effect of the inert gas extinguishing agent is mainly affected by the space sealing and injection flow rate. Based on the above analysis, the design flow rate increases with the opening area will brings better space inerting effect, the larger design flow rate overcomes the negative effects of CO_2_ may leakage from windows. Therefore, under the boundary constraint condition of ventilation opening at a height of 2.45 m, the effect of LCO_2_ on space inerting is more influenced by injection flow than by space tightness. However, a low oxygen concentration may threaten the safety of the staff at the liquor warehouse. When the oxygen concentration is less than 14%, the human body’s activity is reduced, the judgment ability is rapidly reduced, and it is difficult to escape from a burning environment. Under ventilation condition 2, the average oxygen concentration at the space center height of 1.3–1.9 m reaches 14%, which takes approximately 14 s. It only takes approximately 5 s under high injection flow (ventilation condition 3). Therefore, to avoid a suffocation accident of on-site staff, the safety manager should determine the minimum suffocation safety escape time in advance by combining with the design flow and ventilation conditions of the LCO_2_ fire extinguishing system, and formulate an emergency plan. When a fire breaks out, it is recommended that on-site staff should evacuate from the fire site as soon as possible within 20 s between the fire detector activation and the fire extinguishing system activation. Personnel who cannot leave immediately should stay away from the nozzle and quickly evacuate after wearing an oxygen respirator. After all the personnel leave the fire site space, all openings other than the pressure relief opening should be closed to realize the fire site enclosed inerting. Moreover, it must be ensured that the design concentration of the inert gas extinguishing agent in the space is maintained for a specific duration to realize the safe and efficient fire extinguishing of the jar liquor warehouse when using the LCO_2_ system.

### Variation in the space temperature

**Figure 5:**
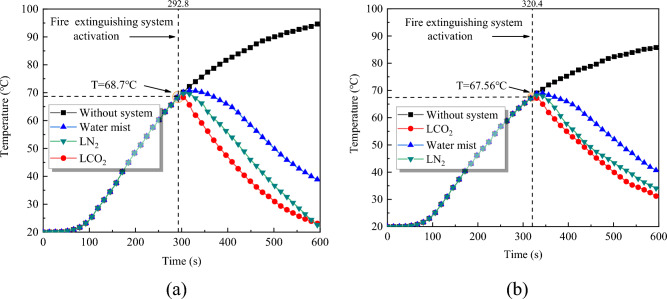
Space temperature change curve at Z = 2.8m. (**a**) Condition 2-I (Normally open-1.8m^2^/jar mouth fire). (**b**) Condition 3-I (Normally open-3.2m2/jar mouth fire).

The flash point of liquor is low, and the space temperature significantly influences the evaporation of ethanol in liquor. Figure 5 shows the space temperature change curve obtained from the heat detector under the jar mouth fire. Clearly, within 20 s after the heat detector reaches 65 °C, the temperature detected by the heat detector changes little. The temperature increases to 68.7 °C, 67.56 °C under Conditions 2-I and 3-I, respectively. The temperature rise range within 20s is less than 5 °C, which is significantly slower than the temperature rise rate under the pool fire (the temperature of Condition 3-II rises to 101.1 °C). This can be used to judge the fire source type in the investigation of fire accidents in liquor warehouses.

After the activation of the fire extinguishing system, the temperature decreased effectively, with the highest decrease noted when using the LCO_2_ system. Under Condition 2-I, the temperature under the action of the LCO_2_ and LN_2_ fire extinguishing systems decreased to approximately 22 °C at *t* = 600 s; however, when the opening area increased to 3.2 m^2^, the cooling rates of these systems decreased, and the temperature decreased to approximately 35 °C at 600 s, whereas the water mist system was almost unaffected. This is because under Condition 3-I, the opening area increased, and the lowest position of the window decreased to 2.45 m. At this time, after the fire source is extinguished, LCO_2_ and LN_2_ are more likely to exchange heat with the external air. At a height of 2.8 m and at a higher position of the ceiling, the flue gas temperature dropped gradually, while the temperature of the water mist was 20 °C. The water mist is mainly aimed at cooling the area below the nozzle, particularly after the fire source is extinguished, and it cannot move upward like the inert gas. Therefore, its direct control ability for high-temperature smoke in the ceiling is limited.

**Figure 6:**
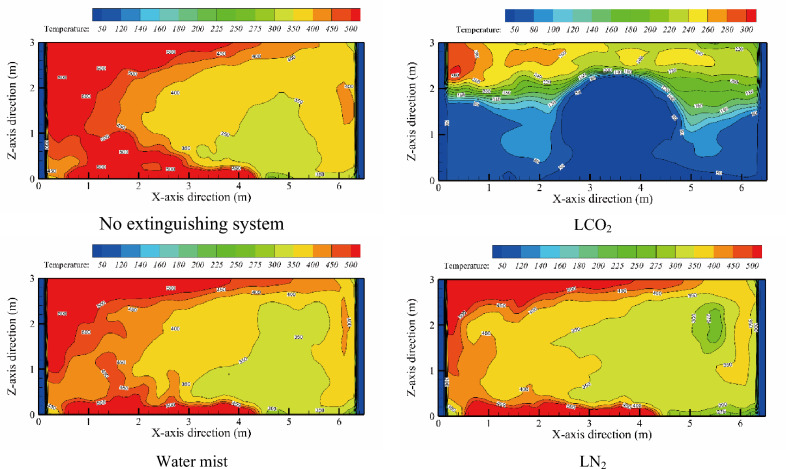
Temperature slices at Y = 3m under different fire extinguishing systems.

As mentioned in “Variation in the oxygen concentration at the bottom of the space” section, the greater the fire source area, the less noticeable the ability of the fire extinguishing system to replace oxygen. When the fire source area was large, the space temperature increased faster, and the fire risk in the liquor warehouse was high. To further analyze the cooling effect of the fire extinguishing system on the high-temperature space, the space temperature change at *Y* = 3 m under Condition 5-IV (open first, then close-3.2 m^2^/pool fire-12 m^2^) was selected (as shown in Fig. 6), and time *t* = 88 s (the fire extinguishing system starts at 10 s) was selected. Since the fire source was located in the X-direction of 0.5 m to 4.5 m, under the action of high pressure, the smoke will flow upward along the left wall, and a thick layer of smoke is accumulated at the top corner. In the X-direction 0–3 m, a high-temperature zone with a temperature higher than 500 °C was formed. Evident ceiling jet phenomenon appeared in the flue gas layer, and a sub-high temperature zone of approximately 400 °C was formed in the X-direction 5–6.5 m. After the fire extinguishing system was applied, the fire source could be effectively suppressed, and the temperature in the space could be reduced. LCO_2_ exhibited the best performance. The fire source was extinguished at *t* = 88 s, and the middle and lower zones (*Z* = 1.5 below) were cooled to less than 100 °C. At this time, in the water mist system, flame cooling is taken as the main fire extinguishing mechanism. When the water droplets encounter a high-temperature flame, they absorb heat and reduce the flame temperature, reduce the convection and radiation between the hot smoke and the liquid surface, and reduce the fire above the surface of the fire source. Under a high oxygen consumption, the fire source is finally extinguished. In comparison, LN_2_ produces a better cooling effect on the flame temperature than water mist and has a better inhibiting effect on fire source development.

## Evaluation of the fire extinguishing performance of different fire extinguishing agents

**Figure 7:**
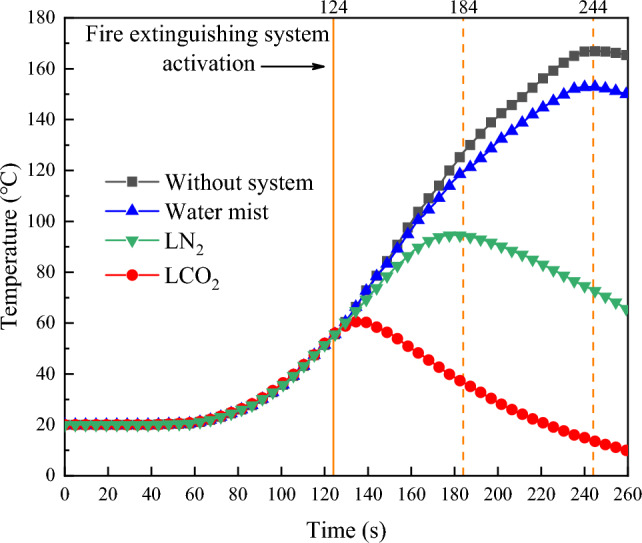
Temperature change curve of space center Z = 1m.

In previous studies, it has been generally agreed that water mist fire extinguishing systems are more effective than CO_2_, N_2_, and other gas fire extinguishing systems under ventilation conditions^35^. According to Dundas’ statistics^40^, when the protective gas leaks from the vent, the fire extinguishing failure rate of the fully submerged halon or CO_2_ system will be as high as 37%. However, through research on the fire extinguishing effect of LCO_2_ and LN_2_ under natural ventilation environment (ventilation conditions 2 and 3), it was found that the success rate of LCO_2_ and LN_2_ was 100%, which is higher than that of the water mist (87.5%). The following are the reasons for the inconsistency in the results between this study and the previous study:

1. In this study, the research subject was liquid inert gas, not the conventional CO_2_ and N_2_ inert gas. Liquid inert gas, in addition to having the function of reducing the oxygen concentration, also has a strong endothermic vaporization cooling ability. Figure 7 shows the temperature change curve of the heat detector at the center of the space *Z* = 1 m, in which the temperature drops the fastest after LCO_2_ injection. According to the calculation of the cooling rate by Eq. (10), the average cooling rates of LCO_2_, LN_2_, and water mist within 60 s of start-up were 1.51, 0.55, and 0.13 °C/s, respectively. The average cooling rates within 120 s were 1.27, 0.78, and 0.12 °C/s, respectively. The liquid inert gas can reduce the space temperature faster and improve the fire extinguishing efficiency than the water mist fire extinguishing system.
  10 $$R_{t} = \frac{{T_{no} (t) - T_{agent} (t)}}{t}$$

**Figure 8:**
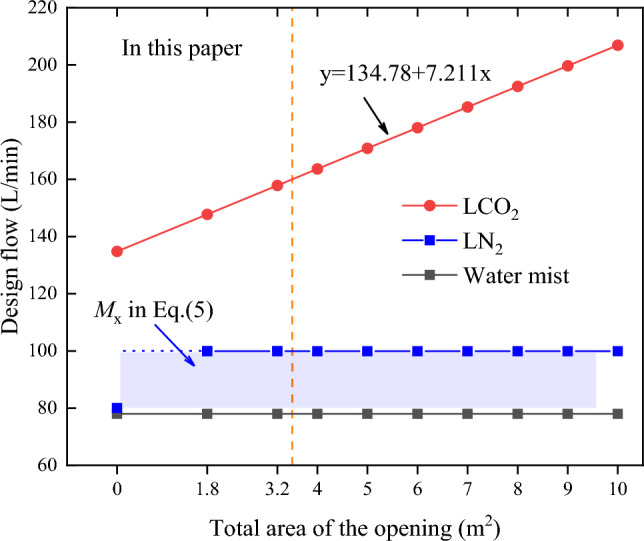
The design flow of fire extinguishing system varies with the opening area.

Here, *R*_*t*_ is the cooling rate in t time, °C/s; *T*_no_ (t) is the temperature at time t without the fire extinguishing system, °C; *T*_agent_ (t) is the temperature at time t under different fire extinguishing systems, °C;

(2) The objective of this study was different from previous studies. Most of the aforementioned scholars studied the influence of ventilation environment on the characteristics of the water mist, considering the fire extinguishing effect of the water mist under a certain condition, with the purpose of finding the optimal parameters for the water mist system. In view of the possible ventilation conditions and fire sources occurring in a pottery jar wine warehouse, in this study, the fire extinguishing time, oxygen isolation, and temperature control effects of low-pressure water mist, LCO_2_, and LN_2_ fire extinguishing systems were compared under different fire simulation conditions through computational fluid dynamics numerical simulation, and the fire extinguishing feasibility, efficiency and cleanliness of the three fire extinguishing systems were comprehensively evaluated.
(3) In addition to the properties of the fire extinguishing agent analyzed above, another key factor affecting the fire extinguishing system efficiency under ventilation conditions is the injection amount of the fire extinguishing agent. Typically, the mathematical relationship between the design flow of the fire extinguishing system and the area of the opening affects the fire extinguishing efficiency under the ventilation condition. Figure 8 shows the design flow of the three fire extinguishing systems under different opening areas. The design flow of the water mist fire extinguishing system is only related to the protected area and the type of protected object^30^, and the influence of the opening area is not considered. The water mist fire extinguishing system was designed with a constant flow rate of 78 L/min under different total opening areas, so that under the same space protection area, the area of the ventilation opening increases, because of which the fire extinguishing performance of the water mist is reduced or even worse, the fire extinguishing may fail (Condition 3-II). Although the LN_2_ fire extinguishing system considers the opening compensation^33^, the compensation amount is independent of the opening area and is not restricted by the room tightness condition. Therefore, it is difficult to ensure the fire extinguishing efficiency of the designed flow under different areas of the opening ventilation. The design flow rate of the LCO_2_ fire extinguishing system varies dynamically with the opening area^34^, thus ensuring an efficient fire extinguishing performance under natural ventilation conditions.

To clarify the feasibility of clean and efficient fire extinguishing by the three fire extinguishing systems in the actual engineering application of the pottery jar liquor warehouse, in this study, six evaluation indices, namely the extinguishment success rate, extinguishment efficiency, cool capacity, oxygen displacement, low cargo damage, and low cost, were established.

The fire extinguishment performance is an important index to evaluate the fire extinguishing system. Based on the principle of the three elements of combustion, this paper concludes that the performance of a fire extinguishing system should be evaluated not only from the results of the fire extinguishment success rate and extinguishment efficiency but also in terms of the oxygen concentration and temperature change in the space.

The fire extinguishing mechanism of the water mist fire extinguishing system varies significantly under different conditions. For example, surface cooling of the fuel is the main fire extinguishing mechanism in the case of a jar mouth fire. Although flame cooling is the main mechanism of pool fire, the large-scale pool fire (12 m^2^) can extinguish the fire quickly by relying on the large amount of space oxygen consumed by the fire source. However, when the pool fire is small (2 m^2^), the effectiveness of the fire is affected by many factors. In this study, the main factor that was expected to result in the low efficiency of the water mist fire extinguishing system was the ventilation condition. The fire extinguishing effect of the water mist fire extinguishing system in a natural environment is not ideal, which is consistent with the conclusion drawn in previous studies in that water mist is affected by natural ventilation^41^ and can withstand natural wind with a certain opening area^42^. However, evidently, under Condition 3-II, the influence limit of the area of opening of the natural ventilation that the water mist can withstand is exceeded, resulting in a fire extinguishing failure. The main reason is that the low water spray momentum generated by the low-pressure water mist system cannot effectively prevent outdoor oxygen from penetrating into the bottom of the space, while the size and location of the fire source will affect the fire extinguishing conditions of the water mist, resulting in a prolonged fire extinguishing time^43^, or even failure. This can also result in the lowest fire extinguishing success rate for the three fire extinguishing systems, at 95%.

Both the LCO_2_ and LN_2_ fire extinguishing systems take asphyxiation and cooling as the main fire extinguishing mechanism. In the initial stage of the fire extinguishing system, the diffusion of the inert gas has a delayed effect on the suppression of the jar mouth fire. However, with the continuous injection of low-temperature inert gas, the fire source is effectively extinguished. The fire extinguishing success rate of the LCO_2_ and LN_2_ fire extinguishing systems reached 100%. After the fire extinguishing system is started, the low-temperature inert gas will spread in the space, which can effectively reduce the space temperature. The latent heat of vaporization of LCO_2_ was 320.4 kJ/kg (− 40 °C), and the latent heat of vaporization of LN_2_ was 199.176 kJ/kg (− 196 °C). The cooling space effect of the LCO_2_ fire extinguishing system is better than that of LN_2_ fire extinguishing system. Compared with the other major gases in the space (such as CO_2_ and O_2_), N_2_ has a lower weight and tends to migrate to the middle and upper parts of the space. Therefore, the LN_2_ fire extinguishing system showed a relatively evident fluctuation in reducing the O_2_ concentration at the bottom of the space. The CO_2_ in the LCO_2_ fire extinguishing system is more likely to accumulate at the bottom of the space, owing to which the LCO_2_ fire extinguishing system outperforms its LN_2_ counterpart in displacing the oxygen concentration at the bottom of the space.

In addition to the above four indices used to evaluate the fire extinguishment performance, low cargo damage and low cost are other important indicators to evaluate the feasibility of the fire extinguishing system. The inert gas permeates the air in the liquor warehouse and will not pollute the liquor. However, compared with an inert gas, the mixing of the water mist and liquor affects the liquor quality and causes secondary damage. Meanwhile, the cold water mist in the high-temperature jar is vulnerable to the impact of thermal stress, resulting in a rupture of the liquor bottle, increasing the expansion of the flowing fire and liquor loss. Therefore, the low cargo damage of the water mist is not as good as LCO_2_ and LN_2_. Cost is an important index to measure the application value of a fire extinguishing system. The installation, use, and maintenance costs of the LCO_2_ and LN_2_ fire extinguishing systems are high, and in terms of the price of the fire extinguishing agent, the cost of LCO_2_ and LN_2_ is roughly in the range of 400–800 RMB/t, and the cost of water mist is also the lowest. However, compared with the market value of liquor in the liquor warehouse, for example, the fireproof zone studied herein can store 20–25 wine tanks (a single wine tank can store 2500 L of liquor), and the total value of the liquor in the fireproof zone is related to its quality type. Taking the domestic medium- and high-end liquor as an example, the market price of a single fireproof zone is 10 million yuan or more. The safety production value brought by LCO_2_ and LN_2_ fire extinguishing systems meets the requirements of economic benefits.

**Figure 9:**
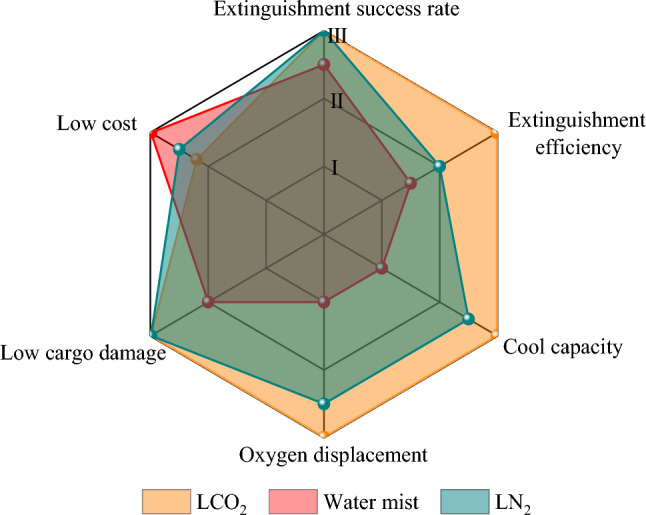
Performance evaluation of fire extinguishing system.

Based on the simulation results and analysis, the performance of clean and efficient fire extinguishing systems for pottery jar liquor warehouses was systematically evaluated. As shown in Fig. 9, the LCO_2_ fire extinguishing system exhibited the best fire extinguishment performance, followed by the LN_2_ system, and lastly the water mist. Both LCO_2_ and LN_2_ outperformed the water mist in terms of the flow cargo damage index. Although the LCO_2_ and LN_2_ fire extinguishing systems are slightly costlier, they are economically beneficial. The feasibility of the clean and efficient fire extinguishing system with LCO_2_ for pottery jar liquor warehouses was confirmed from the comprehensive evaluation results. In the future, the effects of ventilation conditions and fire source type on the fire extinguishing effect and the mechanism of LCO_2_ fire extinguishment will be discussed. These studies will provide a foundation for the future application of the clean and efficient LCO_2_ fire extinguishing system in pottery jar liquor warehouses.

## Conclusions

Based on the numerical simulation experiments of the fire extinguishment process under multiple conditions, the fire extinguishing capability of clean fire extinguishing systems (water mist, LCO_2_, and LN_2_) for liquor warehouses were analyzed in terms of the extinction condition, fire extinguishing time, oxygen concentration, and space temperature. The characteristics and performance of the three fire extinguishing systems were comprehensively evaluated. The aim was to find an optimal clean and efficient fire extinguishing system for pottery jar liquor warehouses. The main conclusions of the study can be summarized as follows:

1. The firefighting results showed that the LCO_2_ and LN_2_ systems exhibited the highest firefighting success rate (100%). The fire extinguishing success rate of the water mist was 95%, and the success rates of the three fire extinguishing systems were all higher than that (45%) under the action of the no-fire extinguishing system, thus confirming the feasibility in extinguishing fires in pottery jar liquor warehouses.
2. The ventilation conditions and fire source type influenced the extinguishing time. Under natural ventilation conditions, the LCO_2_ system required the shortest fire extinguishing time and was almost unaffected by ventilation. However, the water mist system was significantly affected by the ventilation, and the fire extinguishing time was the longest, and the fire extinguishing even failed. For the jar mouth fire, the extinguishing time followed the order (long to short): LN_2_, water mist, and LCO_2_. For the pool fire, the order was water mist, LN_2_, and LCO_2_.
3. In terms of the performance of reducing the displaced oxygen concentration at the bottom of the space and cooling space temperature, the LCO_2_ fire extinguishing system exhibited the best effect, followed by LN_2_, and lastly the water mist. LCO_2_ and LN_2_ were found to be superior to the water mist in terms of the firefighting cleanliness for pottery jar liquor warehouses. In terms of the six evaluation indices, the LCO_2_ fire extinguishing system exhibited excellent characteristics such as cleanliness and high efficiency for pottery jar liquor warehouses.

## Data Availability

All data generated or analysed during this study are included in this published article.
